# Tracing Crustal and Anthropogenic Sources of Metal(loid)s in Hurricane Harvey Floodwater Remnants in Houston, Texas

**DOI:** 10.1021/acsestwater.5c01443

**Published:** 2026-03-05

**Authors:** Sourav Das, Vikram Kapoor, Shankararaman Chellam

**Affiliations:** † Department of Civil and Environmental Engineering, Texas A&M University, College Station, Texas 77843-3136, United States; ‡ School of Civil & Environmental Engineering, and Construction Management, 12346 The University of Texas at San Antonio, San Antonio, Texas 78249, United States; § Department of Chemical Engineering, Texas A&M University, College Station, Texas 77843-3122, United States

**Keywords:** extreme events, stormwater, rare earth elements, vehicular emissions, built environment, natural disasters

## Abstract

Probable sources of metal­(loid) contamination in Hurricane Harvey floodwater remnants from diverse land use settings in Houston, Texas, were investigated. The primary novelties of this work are that we (i) analyzed a wide suite of 51 elements, including rare earths and (ii) implemented two independent multivariate statistical techniques to obtain clues to metal­(loid) sources. This approach differs from many previous studies that simply reported the concentrations of a limited number of metals in hurricane floodwaters. Hierarchical cluster analysis and principal component analysis both resolved three major and statistically distinct source categories: crustal materials, vehicular emissions, and the built environment. The crustal source was confirmed using light rare earth ternary diagrams, yttrium/holmium ratios, cerium and europium anomalies, and Oddo-Harkins patterns. The influence of motor vehicles and traffic was identified using enrichment factors and simultaneous barium–cadmium–antimony and gallium–cadmium–antimony three-component variations. Efflux from the built environment was validated via signature elemental ratios and zinc–tin–lead ternary variations. Overall, the deluge appears to have mobilized metal­(loid)­s with vehicular residues and building materials contributing substantially to floodwater contamination beyond natural crustal dissolution. The source attribution framework developed herein provides a generalized approach to identify metal­(loid) sources in any flood-prone urban environment.

## Graphical abstract

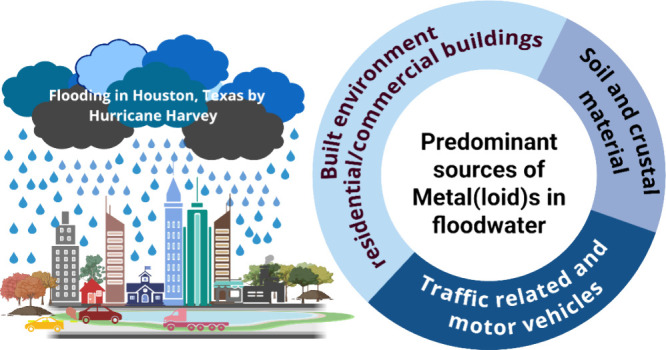

## 1 Introduction

Hurricanes and tropical storms pose significant threats to coastal communities through both immediate physical damage and longer-term environmental contamination. In August 2017, Hurricane Harvey made landfall along the Texas coast as a Category 4 hurricane causing catastrophic flooding in the Houston metropolitan area where it stalled and dropped 36–48 in. of rain over a three-day period.^1,2^ It ranks first in terms of the peak value and the spatial extent of rainfall recorded in the ∼ 135 years since recordkeeping began.^2^ In Harris County alone (where Houston is located), approximately 177,000 homes and buildings were flooded, representing 12% of all structures in the county.^3^ The widespread inundation overwhelmed municipal infrastructure, releasing untreated or partially treated wastewater from more than 800 treatment plants and numerous sanitary sewer overflows.^4^ The unparalleled flooding also raised concerns about potential loss of containment integrity at 13 active Superfund sites in Harris County containing organics and metal­(loid)­s as well as spills and leaks from the region’s extensive network of metal manufacturing, recycling, and handling facilities and one of the world’s largest petrochemical complexes.^3,5^

Previous investigations of hurricane-impacted areas have documented the mobilization and redistribution of chemical and biological contaminants through floodwaters.^6–8^ Following Hurricanes Katrina and Rita in 2005, studies in New Orleans, Louisiana revealed elevated concentrations of lead, arsenic, and other metals in soils and sediments deposited by floodwaters.^6,9,10^ Metal­(loid)­s persisted in soils and sediments for extended periods with little change observed between sampling campaigns conducted months apart, suggesting their strong binding and limited natural attenuation.^6^ Arsenic and lead levels were significantly elevated near industrial facilities, hazardous waste sites, and major traffic corridors in Houston soils post-Harvey^3,5^ but data from residential communities and less-trafficked corridors are lacking.

While these earlier studies qualitatively documented post-hurricane metal concentrations, some critical knowledge gaps remain: (i) the relative contributions of natural versus anthropogenic sources to flood-mobilized metals remain poorly understood and (ii) source attribution methods have not been systematically applied to urban flood scenarios, despite their success in atmospheric studies.^11,12^ Previous water quality investigations in the aftermath of hurricanes documented metal concentrations but did not distinguish natural weathering from human-derived sources.^5–7,9,10^ Existing stormwater studies largely focused on steady-state conditions and lack extreme event characterization arising from catastrophic flooding that can mobilize metals from different sources.^13–16^ Rare earth elements (REEs), trace metals, and metal­(loid)­s can serve as effective fingerprints for their sources. REEs typically maintain crustal ratios and patterns (e.g., North American Shale Composite, NASC) in natural weathering processes whereas anthropogenic sources such as petroleum refining catalysts and vehicular emissions exhibit distinct REE fractionation patterns.^17,18^ Similarly, ratios such as Zn/Cu, Pb/Cu, and Ba/Sb and simultaneous three component variations in main group elements and transition metals can distinguish between traffic emissions (brake wear, tire abrasion, and tailpipe)^16,19,20^ versus leaching from building materials (galvanized surfaces, painted structures, and plumbing fixtures).^13,21,22^

Houston’s complex urban landscape includes diverse potential sources of metals: crustal materials from soils and sediments as well as anthropogenic emissions from vehicles and road dust from extensive highway networks, industrial facilities including one of the world’s largest petrochemical complexes, and the built environment consisting of residential and commercial buildings with metal-containing infrastructure (roofing, plumbing, painted surfaces).^23,24^ Understanding the relative contributions of these sources is critical for developing targeted mitigation strategies, better understanding elemental cycling (including anthropogenic forcings), and assessing potential environmental degradation and human health risks.

The overarching objective of this research was to obtain clues to the sources of a wide suite of metal­(loid)­s in Hurricane Harvey floodwater remnants across Greater Houston. We measured 51 elements including REEs in floodwater remnants collected from diverse land use categories (residential, commercial, traffic corridors, and water bodies) using inductively coupled plasma – mass spectrometry (ICP-MS) and compared them to background samples collected under nonflooding conditions. Hierarchical cluster analysis (HCA) and principal component analysis (PCA) were employed to resolve the contributions of crustal, vehicular, and built environment sources. REE ratios, ternary diagrams, and enrichment factors validated source assignments and confirmed anthropogenic enrichment. This work addresses three overarching questions. First, what are the plausible sources of metal­(loid)­s dispersed by a hurricane across a large metropolitan area? Second, can REE signatures and diagnostic geochemical ratios distinguish natural crustal versus anthropogenic contributions? Third, what are some underlying spatial patterns of source contributions across a diverse urban landscape? We use Hurricane Harvey and the greater Houston area as representative examples and test bed to answer these questions. Overall, results reported herein add to our knowledge of metal­(loid) concentrations, their origins, and behavior following extreme flooding events in general.

This study is novel in many respects as related to understanding flood-related metal­(loid) contamination. First, we comprehensively fingerprinted 14 REEs (including Y) and combined them with 37 other elements to distinguish crustal and anthropogenic sources in hurricane floodwaters, an approach never before applied to extreme flooding events. Second, we identified broad source categories and validated results through two independent statistical methods viz., HCA and PCA, and also corroborated them using geochemical tracer data (REE patterns, enrichment factors, signature ratios, and ternary diagrams). Third, we demonstrated that REEs in Houston floodwaters originated from natural crustal weathering rather than petroleum refining catalysts, despite the region’s extensive petrochemical industry. Fourth, we developed a quantitative multisource attribution framework that traced metals to crustal dissolution, vehicular emissions, and building infrastructure, and provided spatially resolved information on contamination sources across diverse urban land uses.

## 2 Materials and Methods

### 2.1 Study Area and Sample Collection

Water samples were collected between August 30 and September 5, 2017, soon after travel was permitted by law enforcement authorities. An *a priori* identification of representative sampling locations was not possible because of the considerable logistical complications involving site access and safety concerns. The spatial distribution of samples was attempted to capture representative impacts across Houston’s (sub)­urban landscape. Forty samples were obtained across diverse land-use categories (Figure 1): 13 from residential neighborhoods (including flooded homes), 10 from commercial locations, 5 from sites adjacent to major traffic corridors, 11 from water bodies such as bayous, retention ponds, and lakes, and one representing atmospheric input. An atmospheric sample collected from elevated, open location represents atmospheric deposition, consistent with standard practice.^25^ Although collecting only a single sample limits precision of atmospheric contribution estimates, it does not affect identification of dominant source categories, which was our main focus. Samples were also collected in March 2018, from 12 sites that retained standing water under nonflooding conditions to establish background situations. These samples collected 6 months post-Harvey represent baseline nonflooding conditions rather than immediate pre-storm state, which may be affected by seasonal or hydrological variability. Sampling locations are described in more detail in Section S1 of the Supporting Information (SI).

**Figure 1:**
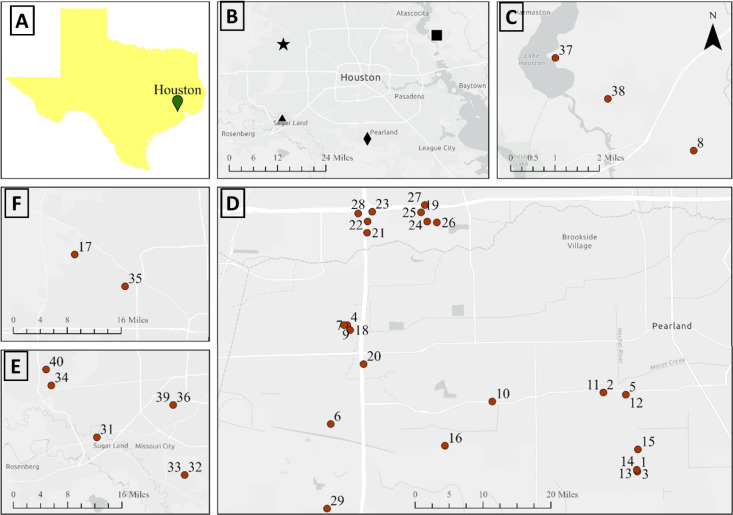
Clockwise from top left. (A) Map of Texas showing Houston. (B) Map of greater Houston showing sampling pockets. (C) Close-up map of sampling locations in the northeast labeled with a square in panel B. (D) Close-up map of sampling locations in the south labeled with a diamond in panel B. (E) Close-up map of sampling locations in the southwest labeled with a triangle in panel B. (F) Close-up map of sampling locations in the northwest labeled with a star in panel B. See Table S1 for detailed sampling information including Harvey floodwater remnants and the background (under nonflooding conditions). All spatial data (and locations) were mapped using the World Geodetic System 1984 (WGS84) geographic coordinate reference system.

Grab samples were collected in precleaned 500 mL polypropylene bottles. Field blanks were included at selected sites to evaluate potential contamination during handling and transportation. Samples were placed on ice immediately following collection, transported to the laboratory within 6 h, and vacuum filtered through sterile 47 mm diameter, 0.2 μm pore size poly­(ether sulfone) membrane filters using acid-washed filtration assemblies in a Class II biosafety cabinet. The filtrate was transferred into acid-washed high-density polyethylene bottles and stored at 4 °C until analysis.

### 2.2 Elemental Analysis: Sample Preparation and Instrument Operation

All filtrates were first acidified to pH < 2 using ultrapure nitric acid (Fisher Optima grade) in accordance with U.S. EPA Method 200.8. Prior to analysis, aliquots were diluted to a final matrix of 1% nitric acid to match calibration standards. Concentrations were measured using a PerkinElmer NexION 300 quadrupole ICP-MS equipped with a dynamic reaction cell (DRC). Internal standards (^115^In and ^209^Bi) were continuously introduced to correct for matrix effects and instrumental drift. Each sample was analyzed at three dilutions (1×, 10×, and 100×) to ensure accurate quantification across the wide dynamic range of concentrations. Fe, Cr, Cu, and Zn were analyzed in DRC mode with ammonia as the reaction gas to reduce polyatomic and isobaric interferences.^26^ A total of 51 main group elements, transition metals, and REEs were analyzed. Calibration standards were prepared from multielement solutions (Antylia Scientific) spanning 0.1–100 μg/L wherein all calibration curves were highly linear (R^2^ > 0.995). More details including quality control are in SI Section S2.

### 2.3 Data Normalization and Statistical Analysis

Elemental concentrations were normalized by electrical conductivity and corroborated using total dissolved solids (TDS) to account for spatially variable dilution associated with extreme precipitation and flooding. In flood-impacted systems, absolute aqueous concentrations can be dominated by dilution effects rather than source-related processes.^27,28^ Normalization by conductivity or TDS reduces this bias and facilitates comparison of relative elemental associations across samples, thereby improving the interpretation of multivariate statistical results. A related approach commonly used to address variable mixing is flow normalization, which accounts for changes in discharge.^28,29^ However, because it was not possible to evaluate rainwater mixing for individual samples, concentrations were normalized by conductivity/TDS and log-transformed to reduce skewness and approximate normality prior to statistical analyses. HCA was performed using Ward’s method and Euclidean distance to group elements with similar covariance patterns. Li, Be, and Na were excluded from HCA. Sodium was removed because of its strong correlation with salinity, which resulted in limited variability and caused it to behave independently from other elemental clusters. Beryllium was excluded because many measurements were below detection limits, whereas Li was removed because a small number of samples exhibited exceptionally high concentrations, leading to disproportionately large distances relative to other elements. PCA with varimax rotation was employed to reduce data dimensionality and to identify dominant geochemical associations among the analyzed elements.^30,31^ Component retention was evaluated using multiple complementary criteria, including cumulative variance explained, inspection of the scree plot, and the Kaiser criterion (eigenvalues >1). The eigenvalues of the first six components were 19.8, 11.2, 6.25, 3.8, 2.1, and 0.63, respectively. Although five components exceeded the Kaiser threshold, the first four components collectively explained more than 90% of the total variance. The scree plot exhibited a clear inflection at the fourth component, and the fifth component contributed <5% additional variance without clear geochemical/environmental interpretability. Accordingly, four principal components (PCs) were retained for further analysis and interpretation. Despite a sampling strategy intended to represent diverse land use settings, the percentage of variance captured by each principal component and their relative ranking may be influenced by the number of sampled environments (see section 2.1 and SI section S1). Statistical analyses were performed using OriginPro 2019b software.

## 3 Results

### 3.1 Elemental Concentrations

All measured concentrations are summarized in SI Figure S2. Li, Na, Ca, Ti, V, Co, Cu, Ni, Zn, Ga, Ge, Se, Sr, Zr, Sb, Ba, Pb, and Eu (as well as conductivity) were almost always lower in concentration in floodwater remnants compared to background values. Al, Si, As, Sn, Sm, Mg, K, Sc, Mn, Cr, Rb, Y, Mo, Cd, Gd, Tb, Dy, Ho, and U were sometimes lower and other times higher in floodwater remnants compared to background values. Only Fe, Nd, and Hf were consistently concentrated in floodwater remnants than their respective backgrounds. Among the metals included in the National Stormwater Quality Database (NSQD),^32,33^ Ni, Cu, Cd, Ba, and Pb exhibited higher concentrations than the average NSQD values; however, none of their measured concentrations were sufficiently high to raise acute toxicity concerns similar to earlier reports from Hurricane Katrina.^6–10^

### 3.2 Hierarchical Cluster Analysis (HCA)

Elements were grouped into four categories based on their correlation and covariation using HCA (Figure 2a). Group 1 consisted of the rare earths, Si, Ca, Mg, and Hf and assigned to crustal materials.^34–37^ Group 2 consisted of Sb, Mo, Cd, Ba, Se, As, Ga, and Zr which primarily originate from motor vehicles and roadside dust.^38–42^ Group 3 did not easily identify with single particular source category but Al, Mn, Fe, Ni, K, Cr, Co, Sn, Zn, Cu, and Pb are well documented to be emitted from anthropogenic (industrial or residential surfaces) sources.^13–16,19,21,22,43,44^ Group 4 was a small cluster consisting of Ti, W, and V and was not identified as belonging to any specific source.

**Figure 2:**
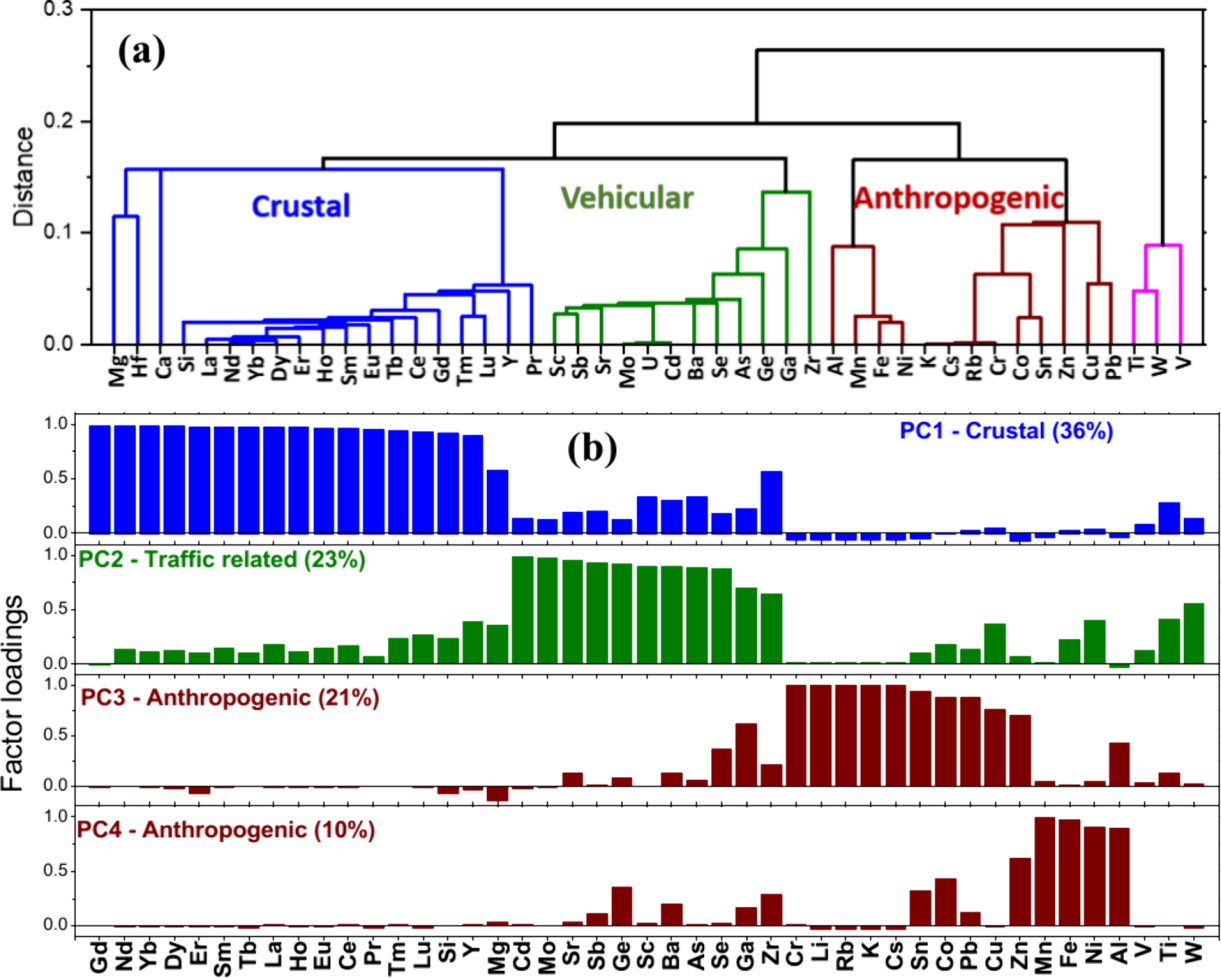
(a) HCA categorized elements into four groups: crustal, vehicular, anthropogenic, and unspecified. (b) PCA resolved four components tagged as crustal, vehicular, and anthropogenic.

### 3.3 Principal Component Analysis (PCA)

PCA with varimax rotation^30,31^ also resolved four major components, capturing 90% of the total variation in the data set (Figure 2b). The first component (PC1) explained 36% of the variation and factor loadings were related to rare earths, Si, and Mg. Importantly, this elemental set overlapped with HCA group 1. The second component (PC2) explained 23% of the variation and factor loadings comprised of Cd, Mo, Sb, Ba, Se, Ga, Zr, and Cu. Because these metals are associated with vehicular emissions and road dust, PC2 was inferred to be similar to HCA group 2. The third and fourth components (PC3 and PC4) explained 23% and 10% of the variations, respectively, and contained all anthropogenic metals that were in HCA group 3. Similar to group 4 in HCA, V, Ti, and W were not strongly affiliated with any single component and exhibited mixed loading distribution among PC1, PC2, and possibly another unresolved component. Based on the elements associated with each component, PC1 was tagged as “crustal,” PC2 as “vehicular,” and PC3 and PC4 as “anthropogenic” originating from multiple industrial sources or residential metal surfaces.^13–16,19,21,22,43^

## 4 Discussion

### 4.1 Principal Component 1 and HCA Group 1 (Crustal Elements, Including Rare Earths)

The first principal component (PC1) explained ∼ 36% of the total variance in elemental concentrations and was dominated by REEs, Si, Mg, Ca, and Hf. The statistical grouping of these elements was consistent with their strong pairwise correlations in both Harvey remnants and the background (Figure 2). This coherence reflects a common source with similar geochemical composition. Comparisons of REE ratios with North American Shale Composite (NASC) values validated their crustal origins. Binary ratios of light rare earth elements (LREEs, La–Sm) such as La/Ce (∼0.46), La/Pr (∼3.9), La/Nd (∼1.0), and La/Sm (∼5.2) closely matched NASC values^14^ and local Houston soils,^18^ confirming that PC1 (and HCA group 1) primarily captured the dissolution and mobilization of material from the upper lithosphere by floodwaters.

Ternary diagrams of light REEs (La–Nd–Pr, La–Nd–Sm, and La–Ce–Sm) further evidenced crustal signatures where floodwater remnants and background samples clustered around the NASC centroid and local soils (Figure 3), similar to prior studies of lanthanides in greater Houston crustal matter.^35,45^ Anthropogenic enrichment was negligible, as none of the samples deviated from the centroid region and plotted far from the three apexes, which are associated with refinery cracking catalysts or traffic.^18,42^

**Figure 3:**
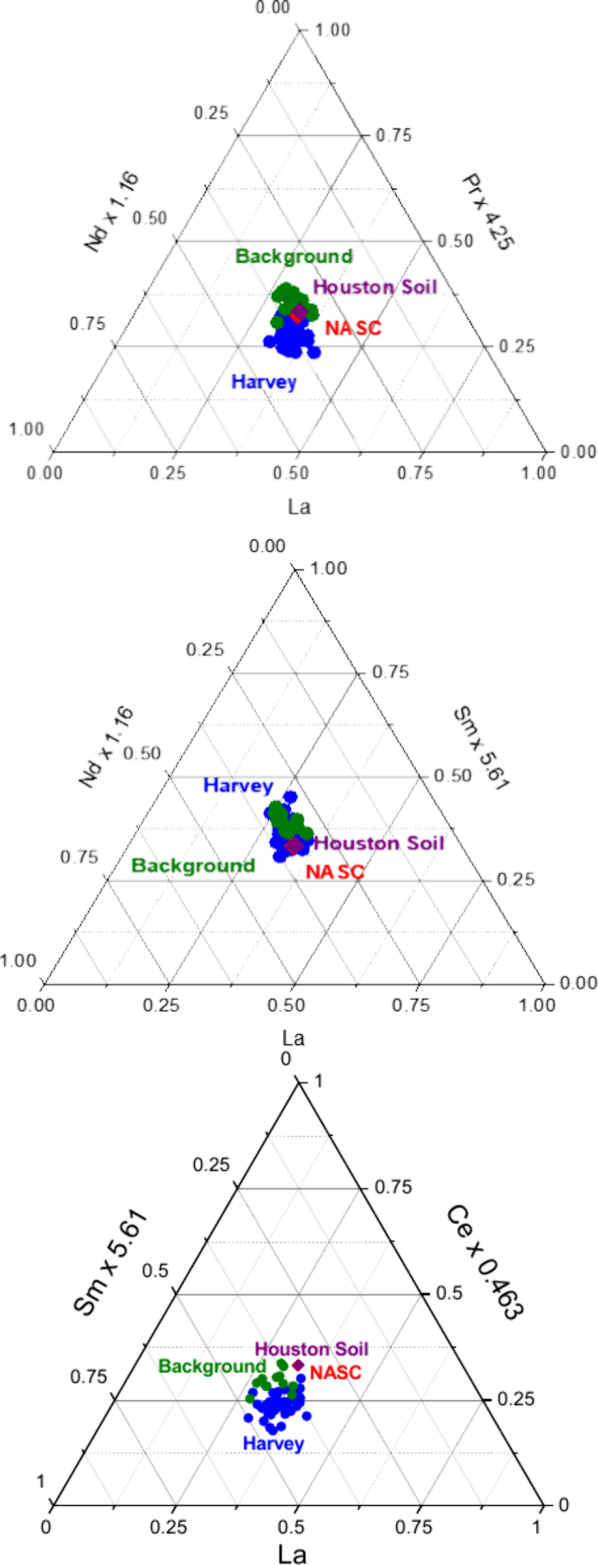
Normalized ternary plots showed clustering of both floodwater and background samples around the NASC centroid. Three component variations in (top) La–Nd–Pr, (middle) La–Nd–Sm, and (bottom) La–Ce–Sm, inferring rare earths in all our samples largely originated from the upper continental crust. Values corresponding to the North American Shale Composite (NASC, red symbol) were used to normalize concentrations to facilitate comparisons, which is why the NASC values plot exactly at the centroid. Blue symbols correspond to Harvey floodwater remnants; green symbols correspond to background samples, and local Houston soil is denoted by the purple symbol. La was used as a base element because it is commonly applied as a representative light rare earth element in crustal studies. Combinations with Ce, Pr, Nd, and Sm were selected because they span the LREE series and provided clear source discrimination. Importantly, we have previously shown that anthropogenic LREEs can be clearly distinguished from natural LREEs in Houston.^12,18,26,35,39,43–45^

Importantly, all Harvey and background samples followed the Oddo-Harkins rule, with even-numbered lanthanides consistently more abundant than their odd-numbered neighbors (SI Figure S4).^11^ This pattern was identical to NASC, upper continental crust, and local soil reinforcing the crustal assignment of PC1.^46^

Further, Ce and Eu anomalies relative to neighboring lanthanides were quantified using the NASC-normalized REE pattern method.^47^ A single previous study has evaluated cerium anomalies pre- and posthurricane because it is a sensitive tracer of redox-controlled REE fractionation and source contributions allowing the evaluation of whether flooding altered fundamental geochemical relationships or introduced new anomalous REE inputs.^48^ Ours is only the second report of such anomalies in floodwaters and we extend earlier work by incorporating Eu as well. In a smooth, undisturbed shale-normalized REE profile, each lanthanide should fall on a predictable trend defined by its neighbors. The corresponding anomalies were expressed as Ce/Ce* and Eu/Eu*:

$$\begin{array}{l} \mathrm{Ce}/{\mathrm{Ce}}^{*}=\frac{{\mathrm{Ce}}_{N}}{\sqrt{\left({\mathrm{La}}_{N}\right)\left({\mathrm{Pr}}_{N}\right)}}\\ \mathrm{Eu}/{\mathrm{Eu}}^{*}=\frac{{\mathrm{Eu}}_{N}}{\sqrt{\left({\mathrm{Sm}}_{N}\right)\left({\mathrm{Gd}}_{N}\right)}} \end{array}$$

where the asterisk (*) denotes theoretical concentrations calculated by geometric interpolation between adjacent lanthanides and the subscript N denotes NASC-normalized concentrations.

Because these ratios represent deviations from the expected crustal pattern, they serve as sensitive indicators of processes that selectively mobilize, oxidize, or scavenge Ce and Eu relative to neighboring REEs. Natural waters usually exhibit negligible anomalies and are governed by weathering, mineral dissolution, and redox transformations; for example, Ce is uniquely sensitive to oxidation (Ce^3+^ → Ce^4+^), and Eu is sensitive to reducing conditions that can stabilize Eu^2+^. In contrast, strong anomalies are typical of anthropogenic sources, such as refinery catalysts enriched in La and Gd^18,45^ or vehicular emissions enriched in Ce and Eu^42^ both of which are important in Houston. Crucially, Ce and Eu anomalies respond strongly to anthropogenic disturbances and therefore are sensitive indicators of rare earth’s origins.^34,49^ REE speciation can be perturbed in industrial discharges, urban runoff, and waste-derived particles, abnormally suppressing or enhancing Ce/Ce* and Eu/Eu* compared to natural waters. In our case, Harvey floodwater remnants displayed only trivial anomalies (Ce/Ce* = 0.80 ± 0.1; Eu/Eu* = 0.98 ± 0.2), lying between the background and Houston soil (or NASC) fields (SI Figure S5). Similar patterns have been attributed to natural weathering and differential leaching.^50,51^ Negligible anomalies measured herein confirm that REEs in Harvey floodwaters were predominantly governed by natural weathering and leaching processes with no evidence for anthropogenic alterations, agreeing with Ce behavior after Hurricane Katrina.^48^

The Y/Ho ratio, a sensitive indicator of fractionation between a light REE (Y) and heavy REEs (Dy–Lu), also distinguished Harvey floodwaters from background samples (SI Figure S6). Background waters exhibited Y/Ho ratios of 24.7 ± 4.5, consistent with Houston soil (28.7 ± 3.4) and NASC (∼27.6).^52,53^ In contrast, Harvey floodwaters were significantly lower, around only half the crustal and background values (13.5 ± 1.6). Since yttrium and holmium are geochemical twins,^53^ this deviation was attributed to differential solubilization during dilution by large volumes of rainwater. Specifically, Ho is more mobile and becomes enriched in the aqueous phase under low salinity and suspended solids conditions, consistent with the lower conductivity (288 μS/cm) and TSS (1,620 mg/L) in Harvey remnants compared to background waters (963 μS/cm and 3,290 mg/L, respectively). This mechanism has been widely reported in studies of freshwater dilution and REE partitioning^54,55^ validating our interpretations.

Signature metal­(loid)­s in the first principal component such as REEs, Si, and Mg quantitatively agreed with characteristic crustal ratios and patterns, indicating that the grouped elements were derived predominantly from surficial soils. Hence, overall, PC1 represented the crustal source in floodwaters. The dominance of crustal sources reflects fundamental geochemical processes during extreme flooding. Hurricane Harvey’s unprecedented rainfall appears to have created conditions for extensive soil-water interaction and mineral dissolution.

### 4.2 Principal Component 2 and HCA Group 2 (Vehicular/Traffic-Related Metals)

The second principal component (PC2) explained ∼ 23% of the total variance and was dominated by metal­(loid)­s typically associated with vehicular emissions and road dust, including Sb, Mo, Cd, Ba, Se, As, Ga, Zr, and Cu.^20,42,56,57^ These elements were strongly intercorrelated (R^2^ generally >0.7; see SI Figure S3), supporting their classification as a single source category. HCA produced a nearly identical grouping (Sb, Mo, Cd, Ba, Se, As, Ga, Zr), further corroborating their common (predominantly vehicular) origin.

To evaluate anthropogenic enhancement of elements associated with PC2, enrichment factors (EFs)^31^ were calculated using a conservative lithogenic reference element (Ti) and a regional aqueous background (Lake Houston). EF analysis is particularly well suited for floodwater systems influenced by variable dilution and source mixing, as it emphasizes relative enrichment patterns rather than absolute concentrations. Titanium was chosen as the datum because it is largely free of anthropogenic influences given the prevalence of petroleum refining, metal working, and recycling industries in Houston that enrich other potential crustal reference elements such as Al, Si, Fe, and Mn in the environment.^42,58^ Further justification of our choice of titanium as the reference element and background can be found in SI Section S6. Figure 4 shows the EFs calculated for vehicular-related metals in floodwaters were substantially elevated. Cd was particularly enriched (reaching as high as ∼ 10,000), consistent with stormwater and roadside studies that identify this metal as one of the most mobile and enriched vehicle-associated metals.^41,59^ Higher enrichment factors of Sb, Ba, Mo, and Zr in near-traffic sites further supported their attribution to brake wear, tire abrasion, and tailpipe emissions.^60–62^ Similar traffic-related enrichments of these elements have been reported in Houston tunnel and roadway dusts^39,42,56^ supporting inferences related to their vehicular origins in floodwater remnants.

**Figure 4:**
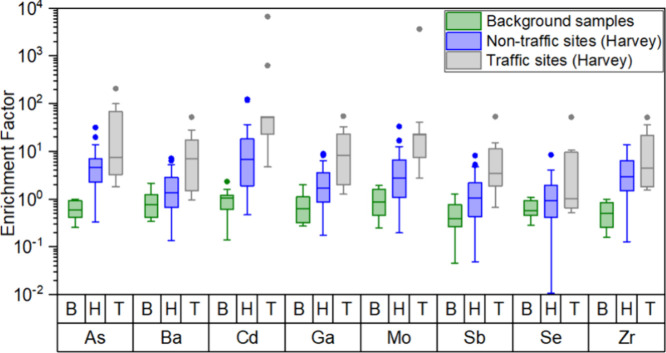
Enrichment factors for various traffic-related metal­(loid)­s shown on a logarithmic scale (using the Ti concentration in Lake Houston as the reference). Green boxes show background samples (denoted by B), blue boxes Harvey remnants from nontraffic locations (denoted by H), and gray boxes Harvey remnants from near-traffic locations (denoted by T). Data depict enrichment of these metals in all floodwater remnants compared to the background, particularly from near traffic sites.

The spatial distinctiveness of PC2 is illustrated in the PC1–PC2 score plot (SI Figure S7). Samples collected near major roadways exhibited strong positive PC2 loadings, clearly separating them from samples collected from residential and commercial locations, and water bodies. This separation underscored the influence of traffic-derived residues, which were preferentially mobilized during flooding.

Simultaneous three component relationships additionally confirmed that PC2 represented vehicular contributions to Harvey floodwaters. In the Ba–Cd–Sb ternary space (Figure 5a), several floodwater samples plotted distinctly toward the Cd and Sb apices, separating them from background and soil endmembers that clustered near the Ba axis. This compositional shift indicated that Cd and Sb, which are strongly associated with brake wear, tire abrasion, and tailpipe particles were preferentially mobilized during flooding.^41,59,60^ In contrast, Ba, a predominantly lithogenic element derived from mineral and crustal sources, exhibited comparatively lower contributions, consistent with minimal soil influence in PC2. The enrichment of Cd and Sb relative to Ba therefore reflects enhanced mobilization of soluble, noncrustal, traffic-derived metal­(loid)­s during the extreme flood event.

**Figure 5:**
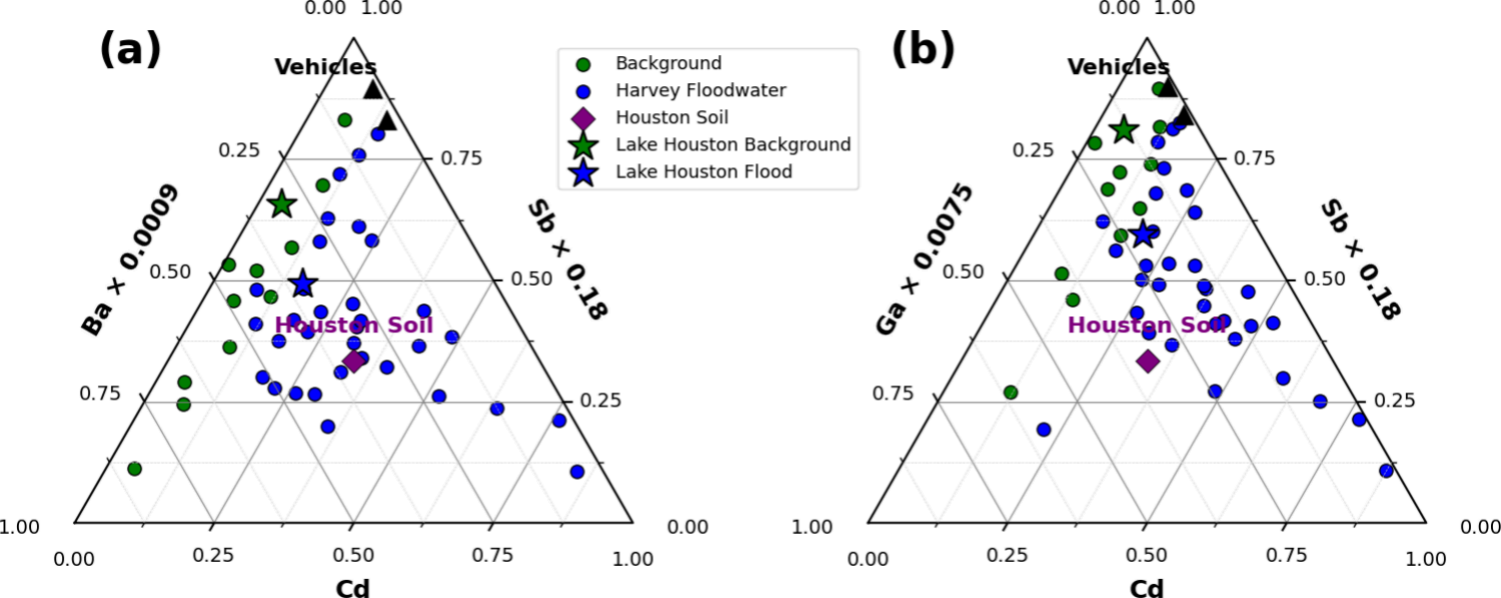
(a) Ba–Cd–Sb and (b) Ga–Cd–Sb ternary diagrams showing anthropogenic enrichment from vehicle emission and road surfaces in Harvey samples relative to local soil. Sb and Cd were selected as traffic-related tracers because they are strongly associated with brake wear and tire abrasion.^20,41^ Ba and Ga were included because they frequently co-occur with Sb and Cd and enhance separation from natural sources in ternary space.^58,63^

The Ga–Cd–Sb ternary diagram (Figure 5b) further reinforced this interpretation. Harvey floodwater samples clustered along the Cd–Sb edge, offset from the Ga-rich (lithogenic) apex, confirming that PC2 metal­(loid)­s largely arose from anthropogenic sources. Ga is well established as a conservative lithogenic element that covaries with Al, Ti, and Fe in natural sediments and is typically used as a crustal tracer.^64,65^ Its relative depletion in Harvey samples, compared with the concurrent enrichment of Cd and Sb, underscored the limited contribution of soil or mineral weathering and the dominant influence of vehicular particulate matter. These compositional displacements across both ternaries in Figure 5 corroborated the enrichment factor and PCA evidence that PC2 reflected mobilization of vehicular residues – particularly brake and tire wear debris – rather than inputs from natural lithogenic sources or stationary urban materials.

Taken together, PC2 strongly captured the mobilization of vehicular metal­(loid)­s during Hurricane Harvey, particularly brake wear residues, tire dust, and road surface materials. Strong enrichment of Cd, Sb, Ba, Mo, and Zr, statistically significant interelement correlations, elevated enrichment factors, spatial clustering near traffic corridors; and shifts in (Ba, Ga)–Cd–Sb ternary diagrams provided compelling evidence that PC2 is a traffic-related component distinct from both crustal inputs (PC1) and other urban anthropogenic sources discussed next (PC3).

### 4.3 Principal Component 3 and HCA Group 3 (General Anthropogenic Metals)

The third principal component (PC3), explaining about 23% of the total variance was dominated by metals characteristic of the built environment that are widely used in urban infrastructure including roofing and gutters (Zn, Cu), plumbing and wiring (Cu, Ni), painted surfaces and electrical wiring (Pb, Sn), and stainless-steel or alloy fixtures (Cr, Ni, Co).^13–16,19,21,22,43,44^ The same suite of elements exhibited strong loadings in PC3, suggesting mobilization of metals from indoors and structural materials during inundation.

The PC1–PC3 score plot (SI Figure S8) showed that residential and commercial floodwater samples exhibited high positive PC3 scores but low PC1 scores, indicating that this component was chemically distinct from the crustal signature represented by PC1. Such an inverse relationship implied that the metals grouped in PC3 originated predominantly from anthropogenic materials within the built environment rather than from mobilization from or dissolution of natural soils.

Compositional ratios among these metals further substantiated this interpretation (Figure 6). The Zn/Cu ratio was 0.91 ± 0.58 in background waters, which nearly doubled to 1.74 ± 0.96 in commercial samples, and increased 7-fold to 6.70 ± 12.49 in residential samples. Likewise, the Sn/Cu ratio was 0.13 ± 0.15 in background, nearly quadrupling to 0.48 ± 0.23 in commercial samples and nearly quintupling to 0.62 ± 1.14 in residential floodwaters. In contrast, the Pb/Cu ratio remained consistently low across Harvey samples (commercial = 0.03 ± 0.01; residential = 0.03 ± 0.03), suggesting only minor leaching from legacy leaded paint or electrical infrastructure. Strong enrichments of Zn/Cu and Sn/Cu ratios in residential floodwaters relative to the background validated the distinctive infrastructure-derived composition of PC3. These ratios clearly distinguished the residential and commercial samples from traffic-influenced and open-water sites, consistent with the enrichment of metals from inundated plumbing, coated surfaces, and household electrical fixtures.

**Figure 6:**
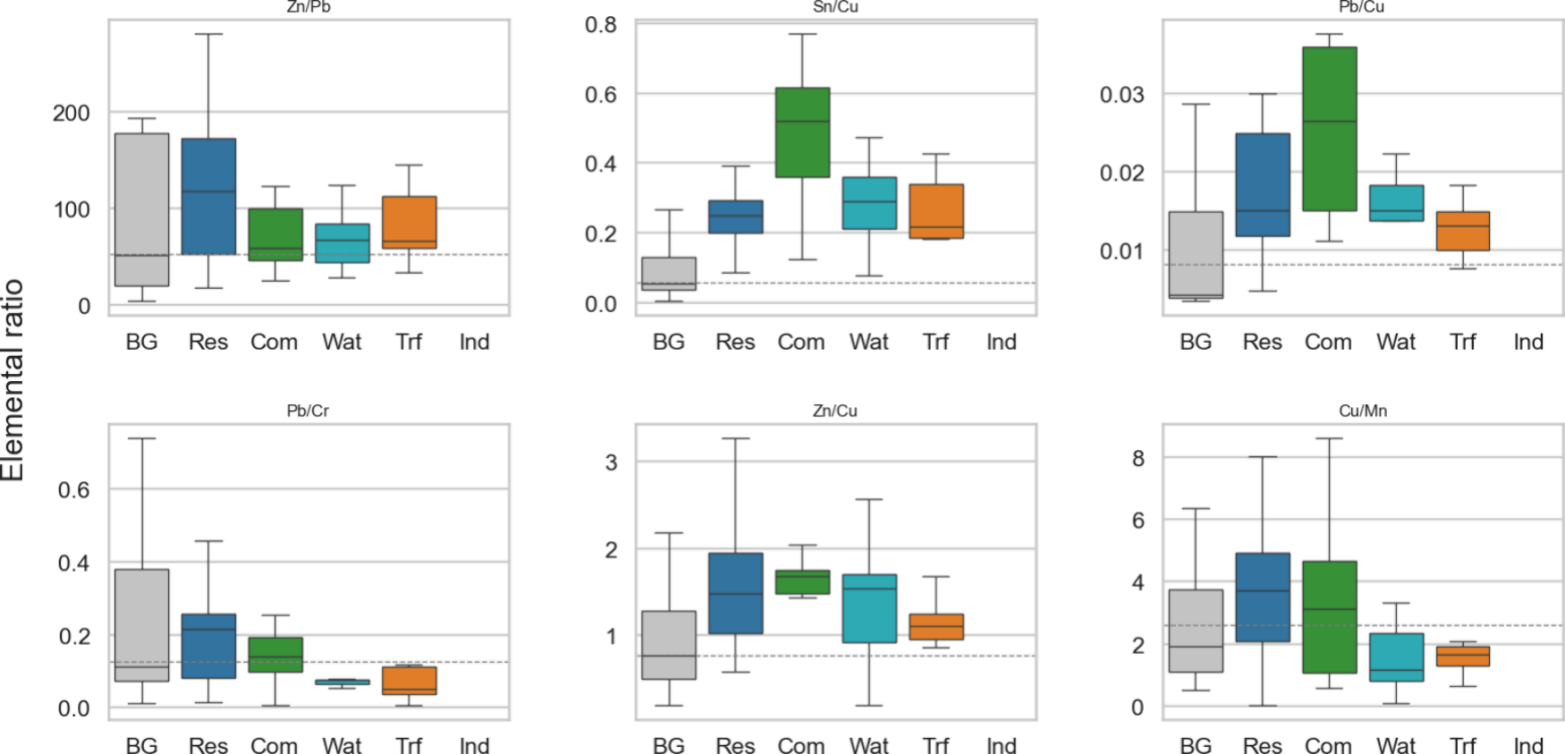
Metallic ratios (clockwise from top left: Zn/Pb, Sn/Cu, Pb/Cu, Cu/Mn, Zn/Cu, and Pb/Cr) of PC3-related metals across sample groups. Boxes show interquartile ranges in background (BG), residential (Res), commercial (Com), waterbody (Wat), and near-traffic (Trf) samples.

Enrichment patterns were further illustrated in the Zn–Sn–Pb ternary diagram (Figure 7), where Harvey floodwater samples clustered toward the Zn axis, distinct from crustal and vehicular end members such as Houston soil, road dust, and airborne particulate matter collected in an underwater tunnel.^42,58^ Compared to vehicular and crustal matrices, which typically exhibit lower Zn/Pb and Sn/Pb ratios, Harvey floodwaters were markedly enriched in Zn and Sn relative to Pb, indicating greater leaching of Zn-bearing coatings, roofing materials, and galvanized surfaces during inundation.^13,14,16,21,69^ Road dust and soil matrices, in contrast, showed proportionally greater contributions of Pb.^39,42,58^ These relative enrichments in Zn and Sn highlighted the influence of leaching from household and infrastructure materials – such as painted or galvanized fixtures and electrical components – during prolonged flooding, further distinguishing PC3 as an urban infrastructure–derived component.

**Figure 7:**
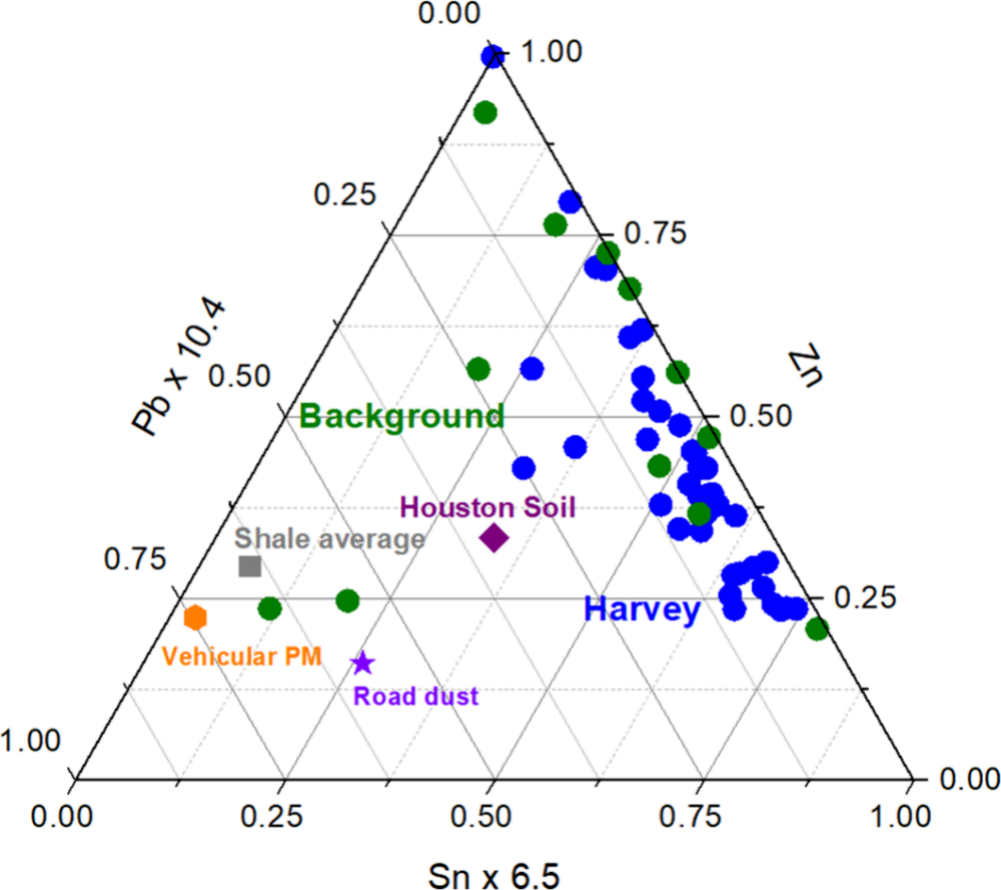
Simultaneous three-component variations in Zn–Sn–Pb showing enrichment patterns in Harvey floodwater remnants. Floodwater samples migrated toward the Zn–Sn axis, distinct from crustal (Houston soil and shale average)^17,42^ and vehicular (road dust, vehicular emissions) end members.^58^ Pb, Zn, and Sn were selected because they are commonly found in household materials, alloys, and building surfaces.^66–68^ This combination resulted in clear separation from traffic-related metals, supporting their interpretation as a distinct anthropogenic source group.

These findings aligned with previous urban runoff and stormwater research that showed dominant Zn, Cu, and Pb fluxes from building materials, whereas Sn, Ni, and Cr frequently traced corrosion from domestic alloys and electrical components.^13,14,16^ Tin, in particular, used extensively in solders and protective coatings, is known to leach under humid or submerged conditions.^69–71^

HCA in Section 3.2 supported this classification, grouping Zn, Cu, Pb, Ni, Cr, Co, and Sn as a distinct anthropogenic cluster separate from the crustal (PC1) and vehicular (PC2) components. Close agreement between HCA and PCA emphasized the robustness of this component’s (PC3) interpretation. Collectively, PC3 represented an indoor and residential infrastructure-derived signature. The elevated Zn/Cu and Sn/Cu ratios, combined with high PC3 loadings in residential and commercial samples, demonstrated that metal leaching from the built environment constituted a significant secondary source of floodwater contamination.

## 5 Conclusions

Previous publications reporting water quality following hurricanes typically only qualitatively documented a limited number of metal­(loid)­s, compared them to acute risk levels, and did not perform any quantitative analyses to obtain clues to their sources.^6,9,18,72–75^ In contrast, this manuscript (i) provided a comprehensive snapshot of the concentrations and plausible origins of numerous main group elements and (inner)­transition metals, (ii) employed two independent robust multivariate statistical techniques to attribute metal­(loid)­s to natural and anthropogenic sources and validated them rigorously with each other and analytical chemistry measurements, (iii) quantified rare earths and obtained strong evidence for their crustal origins rather than cracking catalysts used in petroleum refining,^18,76^ which is a crucial economic activity in Houston, (iv) analyzed 37 non-REEs (i.e., transition metals and main group elements) and traced them back to motor vehicles and building infrastructure components, (v) used low-temperature geochemistry concepts to interpret temporary shifts of the elemental characteristics in an urban watershed caused by a major storm event, which diluted, mobilized, and redistributed metal­(loid)­s from diverse sources, and (vi) developed methods that are directly applicable to other hurricanes and floodwaters without loss of generality.

This comprehensive research effort based on elemental analysis and two independent multivariate statistical techniques identified mobilization of metal­(loid)­s from soils/sediments, motor vehicles, and residential/commercial infrastructure by Hurricane Harvey’s catastrophic flooding. The integrated use of advanced statistical tools, geochemistry concepts, enrichment factor analysis, and ternary compositional diagrams provided robust characterization of metal­(loid) sources in urban floodwaters, revealing that contamination arose from multiple independent routes. To our knowledge, this is the first comprehensive source attribution of metal­(loid)­s in urban floodwater remnants following a major hurricane. The manuscript’s scope did not include the mechanisms controlling metal­(loid) transport, which would require microscale information on soil mineralogy, grain size distribution, organic matter, and sorption processes coupled to macroscale considerations of hydrological dilution, sediment transport, and depositional processes. As climate change and urbanization^1^ increases the frequency and intensity of extreme precipitation events, understanding and managing the environmental consequences of urban flooding becomes increasingly critical for protecting public health and environmental quality. Finally, it is emphasized that this methodological framework can guide post-disaster assessment in other flood-prone cities. Such an approach will enable rapid source screening to distinguish anthropogenic inputs from natural background and guide follow-up studies of metals in the hydrologic system, mobilization pathways, and geochemical behavior during extreme flooding.

## Supplementary Material



## Notes

Details of sampling locations, ICP-MS instrumental settings, method detection limits, quality control aspects, elemental concentrations in floodwater and background samples, enrichment factors, intercorrelations between PC2 metals, details of REE behavior (Oddo-Harkins, Ce and Eu anomalies, and Y/Ho ratios), and score plots between individual principal components (PDF)
