# Supplementary material for: Tracing Crustal and Anthropogenic Sources of Metal(loid)s in Hurricane Harvey Floodwater Remnants in Houston, Texas
Source: ACS ES T Water. 2026 Mar 5;6(3):1955–65. doi: 10.1021/acsestwater.5c01443 (PMC12993842; doi:10.1021/acsestwater.5c01443)
Supplement: Supplementary file 1 [file ew5c01443_si_001.pdf]

SUPPORTING INFORMATION

Tracing Crustal and Anthropogenic Sources of Metal(loid)s in Hurricane Harvey Floodwater Remnants in Houston, Texas

Sourav Das,[a] Vikram Kapoor,[b] Shankararaman Chellam,[a,c,*]

[a] Department of Civil and Environmental Engineering, Texas A&M University, College Station, TX 77843-3136, USA

[b] School of Civil & Environmental Engineering, and Construction Management, University of Texas at San Antonio, San Antonio, TX 78249, USA

[c] Department of Chemical Engineering, Texas A&M University, College Station, TX 77843-3122, USA

[*] Corresponding author: S. Chellam, Zachry Department of Civil & Environmental Engineering, Texas A&M University, College Station, TX 77843. Phone: (979) 458 5914; chellam@tamu.edu

Number of Sections: 8

Number of Figures: 8

Number of Tables: 3

Section S1. Sampling information.

Table S1. Sample description along with location information and the date collected

Samples	Sample number	Type	Latitude	Longitude	Description	Date collected	Collection volume
S1	1	Commercial	29.52274	-95.297785	Temple	Wednesday, 08/30/2017	250 mL + Ziplock
2back - a	2	Background	29.54473	-95.308708	Stella Roberts recycling center (2 Samples)	Tuesday, 03/13/2018	150 mL
2back - b	2	Background	29.54473	-95.308708	Stella Roberts recycling center (2 Samples)	Tuesday, 03/13/2018	150 mL
S2	2	Waterbodies	29.54473	-95.308708	Stella Roberts recycling center (2 Samples)	Wednesday, 08/30/2017	150 mL
S3	3	Commercial	29.52221	-95.29763	Temple Parking Lot (2 Samples)	Wednesday, 08/30/2017	150 mL
S4	4	Residential	29.56413	-95.39217	Pot at Home	Wednesday, 08/30/2017	150 mL
S5	5	Residential	29.5441	-95.301392	Magnolia Elementary (2 Samples)	Thursday, 08/31/2017	150 mL
S6	6	Waterbodies	29.53574	-95.397682	Southfork Lake, Clayton and S. Fork	Thursday, 08/31/2017	150 mL
S7	7	Residential	29.56413	-95.39217	Discovery at Shadow Creek	Thursday, 08/31/2017	150 mL
S8	8	Waterbodies	29.87707	-95.02378	Chevron Gas Station, 2233 FM 1942 Crosby TX, 77532	Thursday, 08/31/2017	1 L
S9	9	Residential	29.56412	-95.393235	Playground	Saturday, 09/02/2017	250 mL
10back	10	Background	29.54216	-95.344906	Southfork Silverlake	Tuesday, 03/13/2018	250 mL
S10	10	Waterbodies	29.54216	-95.344906	Southfork Silverlake	Saturday, 09/02/2017	250 mL
S11	11	Waterbodies	29.54473	-95.308708	Stella Roberts	Saturday, 09/02/2017	250 mL

S12	12	Residential	29.5441	-95.301392	Magnolia Elementary	Saturday, 09/02/2017	250 mL
S13	13	Commercial	29.52274	-95.297785	Temple Roof Leak	Saturday, 09/02/2017	250 mL
S14	14	Commercial	29.52274	-95.297785	Run off Temple Roof	Saturday, 09/02/2017	250 mL
15back	15	Background	29.52855	-95.297455	Pearland bk road Ditch near Warehouse	Tuesday, 03/13/2018	250 mL
S15	15	Road	29.52855	-95.297455	Pearland bk road Ditch near Warehouse	Saturday, 09/02/2017	250 mL
S16	16	Road	29.52959	-95.360421	Kennedy St and Ct Rd 101 near Industry Ditch	Saturday, 09/02/2017	150 mL
17back - a	17	Background	29.92329	-95.697378	Towne Lakes, E. Frio River Circle Marina	Thursday, 03/22/2018	150 mL
17back - b	17	Background	29.92329	-95.697378	Towne Lakes, E. Frio River Circle Marina	Thursday, 03/22/2018	150 mL
S17	17	Waterbodies	29.92329	-95.697378	Towne Lakes, E. Frio River Circle Marina	Saturday, 09/02/2017	150 mL
18back	18	Background	29.5627	-95.391296	Ditch by Apt Complex, Hickory Slough	Tuesday, 03/13/2018	150 mL
S18	18	Residential	29.5627	-95.391296	Ditch by Apt Complex, Hickory Slough	Saturday, 09/02/2017	150 mL
19back	19	Background	29.59638	-95.368121	Fellows Road and Beltway 8 Feeder Industrial	Tuesday, 03/13/2018	150 mL
S19	19	Commercial	29.59638	-95.368121	Fellows Road and Beltway 8 Feeder Industrial	Saturday, 09/02/2017	150 mL
S20	20	Residential	29.55281	-95.386924	288 S @ IHOP and Chuck E Cheese	Saturday, 09/02/2017	150 mL
S21	21	Commercial	29.59049	-95.385749	288 Feeder & Eternity Blvd, St. Thomas Cantebury	Sunday, 09/03/2017	150 mL

S22	22	Road	29.5938	-95.385555	Beltway 8 and Holiday Inn Express and 288 South road runoff	Sunday, 09/03/2017	150 mL
23back	23	Background	29.59656	-95.384067	Culvert on Beltway 8 Feeder & 288 S	Tuesday, 03/13/2018	150 mL
S23	23	Road	29.59656	-95.384067	Culvert on Beltway 8 Feeder & 288 S	Sunday, 09/03/2017	150 mL
S24	24	Waterbodies	29.59375	-95.366113	WWTP/Tom Bass Park retention pond behind industrial location	Sunday, 09/03/2017	150 mL
S25	25	Commercial	29.59637	-95.36812	Culvert behind retention pond, Tom Bass and Beltway	Sunday, 09/03/2017	150 mL
26back	26	Background	29.59351	-95.362948	Drainage channel	Tuesday, 03/13/2018	250 mL
S26	26	Waterbodies	29.59351	-95.362948	Fast moving water on other side of WWTP & Tom Bass	Sunday, 09/03/2017	250 mL
S27	27	Commercial	29.59848	-95.36689	Belt 8/Scott Street. Shell Station	Sunday, 09/03/2017	150 mL
S28	28	Commercial	29.59605	-95.388623	Belt 8 feeder and 288 feeder near Bass Proshop Construction Site	Sunday, 09/03/2017	150 mL
S29	29	Commercial	29.51169	-95.398871	County Road 58 & 288 Manvel, Near Church	Sunday, 09/03/2017	150 mL
S30	30	Waterbodies	30.11303	-94.170545	Discharge point of a detention pond	Sunday, 09/03/2017	150 mL
S31	31	Road	29.61398	-95.65057	Near Highway 6, 90 A intersection	Monday, 09/04/2017	500 mL
S32	32	Residential	29.54403	-95.4633	Ana's House (outside) 722 Linden St (2)	Monday, 09/04/2017	1 L
S33	33	Residential	29.54403	-95.4633	Ana's House (inside flood) 722 Linden 77547	Monday, 09/04/2017	500 mL

34back	34	Background	29.70996	-95.74749	6222 Southcott Ct Katy TX 77450	Wednesday, 03/14/2018	1 L
S34	34	Residential	29.70996	-95.74749	6222 Southcott Ct Katy TX 77450	Monday, 09/04/2017	1 L
S35	35	Residential	29.86391	-95.589152	Ensenada Canyon Lane (2)	Monday, 09/04/2017	500 mL
S36	36	Residential	29.6738	-95.487812	Anat’s house - 5726 Brasheather Drive	Monday, 09/04/2017	500 mL
37back	37	Background	29.94497	-95.13961	Lake Shadows Boat Ramp Water	Wednesday, 03/14/2018	500 mL
S37	37	Waterbodies	29.94497	-95.13961	Lake Shadows Boat Ramp Water	Tuesday, 09/05/2017	500 mL
S38	38	Waterbodies	29.91516	-95.09561	Newport Golf Course Pond Water	Tuesday, 09/05/2017	500 mL
S39	39	Residential	29.6738	-95.487812	Anat’s house - 5726 Brasheather Drive	Tuesday, 09/05/2017	500 mL
S40	40	Residential	29.73968	-95.758523	22310 Morning Lake Dr	Monday, 08/28/2017	500 mL

Section S2. ICP-MS settings, detection limits, and quality controls.

Table S2. Instrumental settings for PerkinElmer® NexION® 300 (DRC-q-ICP-MS). The standard mode refers to the absence of a collision cell gas and DRC mode refers to the use of ammonia as the collision cell gas.

Instrument	PerkinElmer® NexION® 300
Nebulizer	Concentric (Meinhard) [Type A0.5]
Spray chamber	Baffled quartz cyclonic
Torch injector	Quartz
Auto lens	On
RF power	1600 W
Gas flow rates	
Sample	18 L min^{-1}
Nebulizer	0.96-1.18 L min^{-1}
Auxiliary	1-1.2 L min^{-1}
Interface	Platinum cones
Sampler cone	1.1 mm orifice diameter
Skimmer cone	0.9 mm orifice diameter
Measurement parameters	
Scanning mode	Peak hopping
Sweeps/reading	20
Readings/replicate	1
Replicates	3
Dwell time	50 ms (standard mode); 100 ms (DRC mode)
Sampling parameters	AS-93 plus auto-sampler
Sample flush time	45 s
Sample flush pump speed	20 rpm
Read delay	65 s
Read delay pump speed	24 rpm
Wash time	60 s
Wash pump speed	24 rpm
Cell gas	NH_3 (0.2-1.0 mL min^{-1})
RPq	0.25-0.75
RPa	0

Table S3. Method detection limit (MDL) of each element in ICP-MS analysis and mode used for detection

Element	m/z	MDL (μg L^{-1})	Mode	Element	m/z	MDLs (μg L^{-1})	Mode
Li	7	0.03	STD	Mo	96	0.04	STD
Be	9	0.03	STD	Cd	113	0.04	STD
Na	23	0.63	STD	Sn	118	0.04	STD
Mg	25	0.4	STD	Sb	121	0.04	STD
Al	27	0.61	DRC	Cs	133	0.03	STD
Si	29	35	STD	Ba	138	0.22	STD
K	39	1.2	STD	La	139	0.014	STD
Ca	44	11.5	STD	Ce	140	0.015	STD
Sc	45	0.004	STD	Pr	141	0.007	STD
Ti	47	0.22	STD	Nd	146	0.008	STD
V	51	0.79	DRC	Sm	152	0.002	STD
Cr	52	0.1	DRC	Eu	153	0.002	STD
Mn	55	0.03	STD	Gd	158	0.002	STD
Fe	57	1.7	DRC	Tb	159	0.002	STD
Ni	58	0.03	DRC	Dy	164	0.003	STD
Co	59	0.05	STD	Ho	165	0.003	STD
Ge	61	0.014	STD	Er	166	0.003	STD
Cu	65	0.004	DRC	Tm	169	0.002	STD
Zn	66	0.01	DRC	Yb	174	0.002	STD
Ga	69	0.05	STD	Lu	175	0.002	STD
As	75	0.04	DRC	Hf	178	0.001	STD
Se	82	0.11	DRC	W	184	0.04	STD
Rb	85	0.04	STD	Pb	208	0.05	STD
Sr	88	0.04	STD	Th	232	0.012	STD
Y	89	0.03	STD	U	238	0.009	STD
Zr	90	0.04	STD				

STD denotes standard mode

DRC denotes dynamic reaction cell mode with NH_3 as the cell gas

Quality Assurance/Quality Control for metals measurements. ICP-MS measurements were verified using National Institute of Standards and Technology (NIST) Standard Reference Material (SRM) 1643e "Trace Elements in Water." Measured concentrations were compared with certified values in Supporting Information (SI) Figure S3 which agreed quantitatively demonstrating high quality metals measurements. Mid-calibration standards were measured for every ten samples to account for temporal drift as were blank reagents to detect any carryover. Duplicates samples with spiked concentrations were analyzed to account for matrix effects. Another set of duplicate samples were spiked with ruthenium standards to detect signal drift for elements having much lower atomic mass compared to ^{115}In.

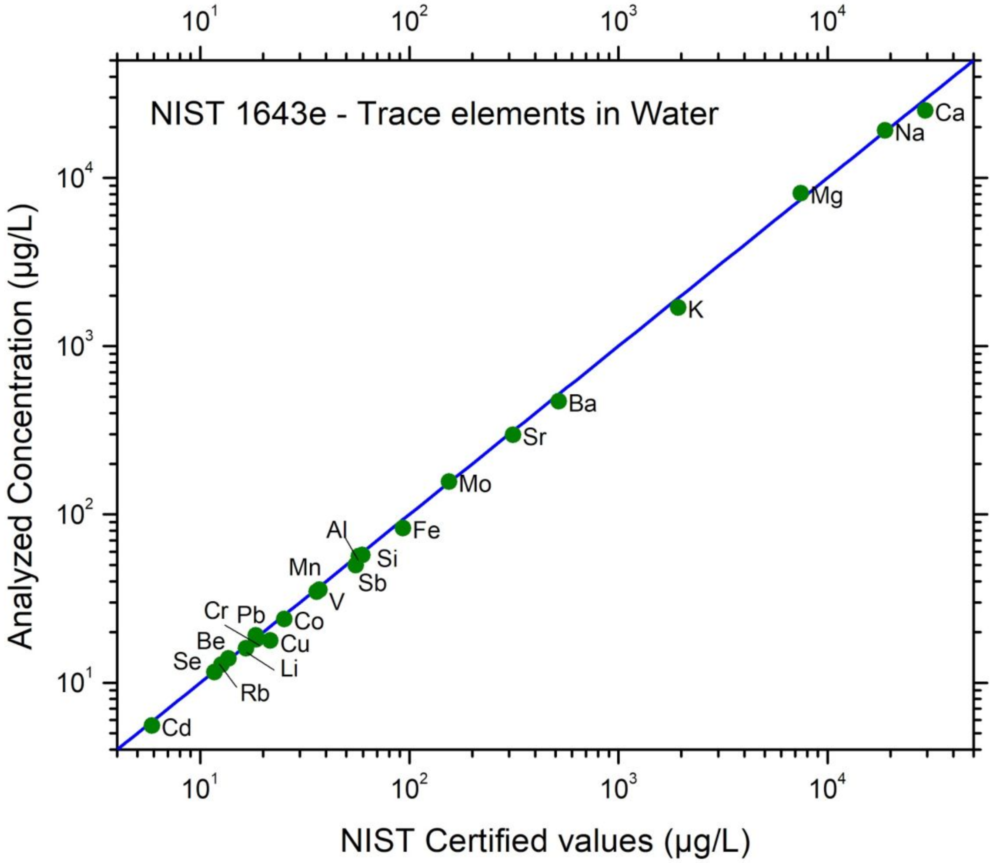

Figure S1. Strong agreement between certified National Institute for Standards and Technology (NIST) Standard Reference Material (SRM) 1643e values and laboratory measurements during this study.

Section S3. Overview of elemental concentrations measured throughout the investigation.

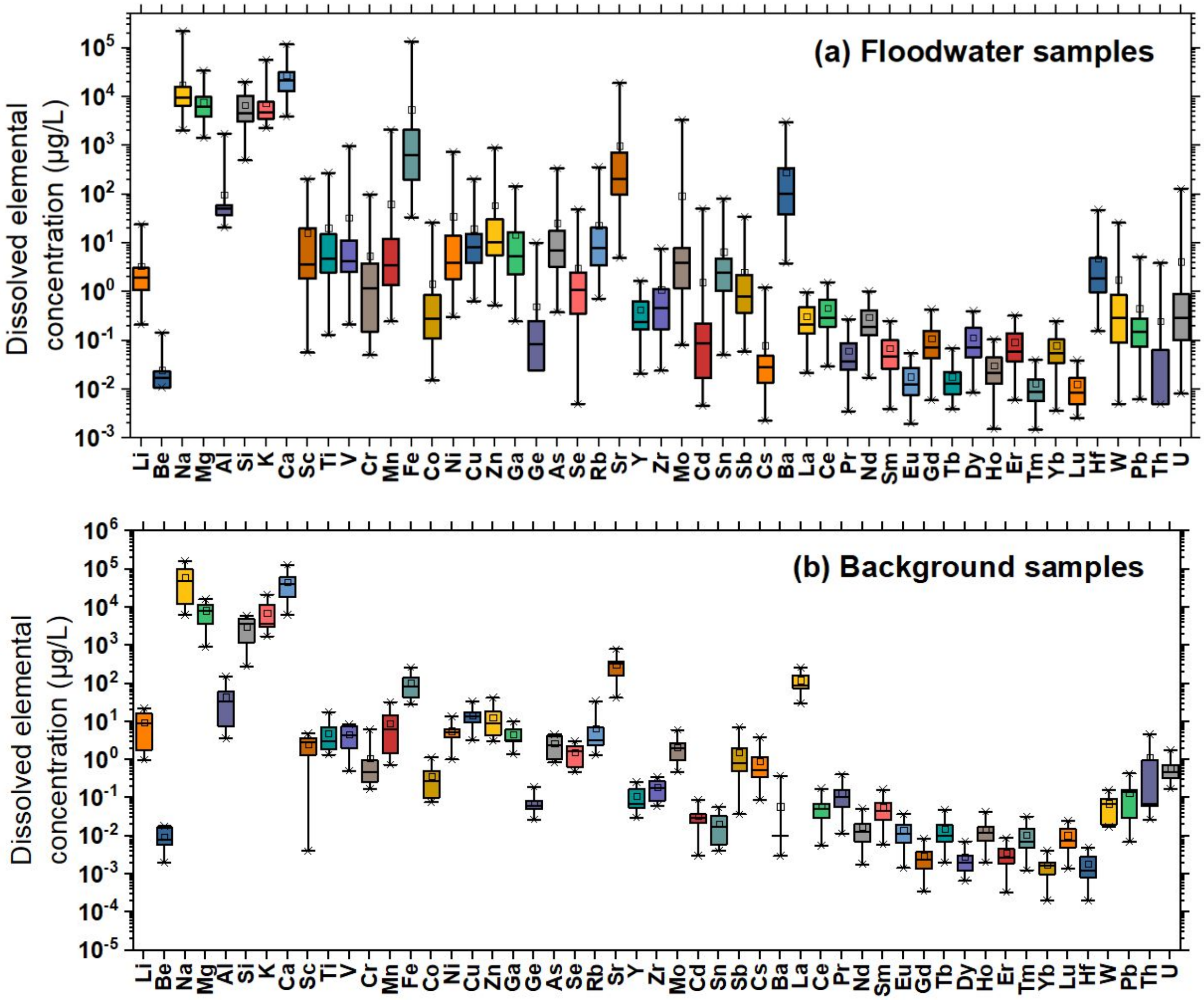

Figure S2. A summary of elemental concentrations in (top panel, a) floodwater and (bottom panel, b) background water samples, respectively.

Section S4. Cross-correlations between traffic associated metals.

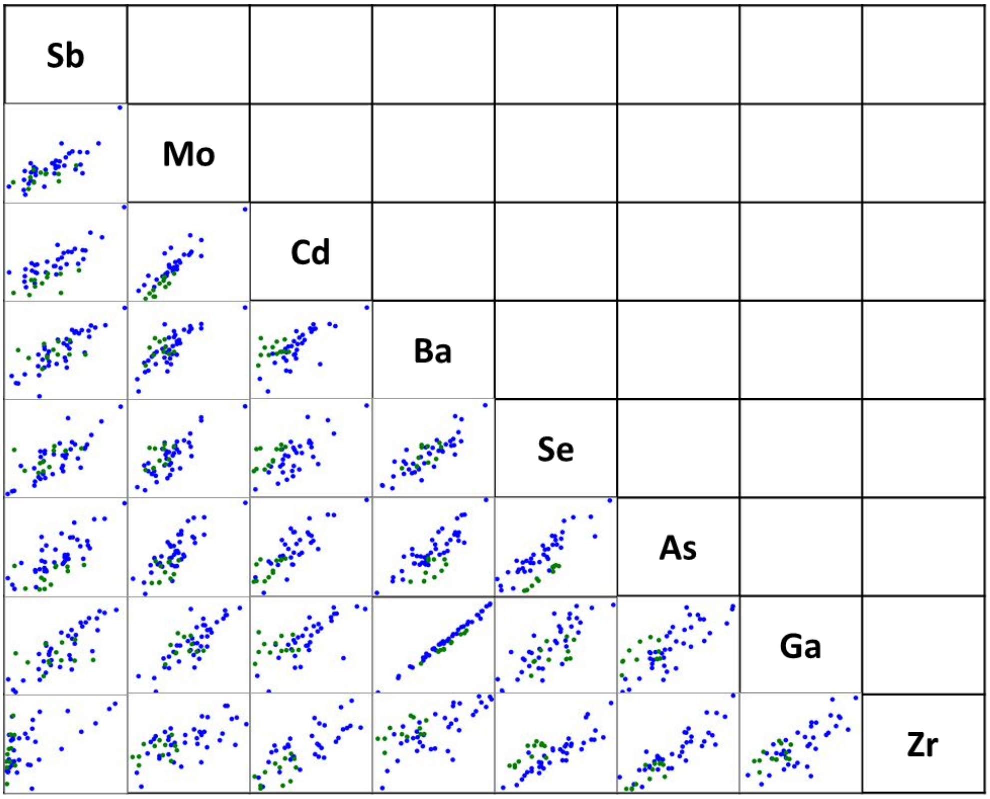

Figure S3. Inter-correlation matrix of traffic-associated metals (Sb, Mo, Cd, Ba, Se, As, Ga, Zr, Cu) in Harvey floodwater remnants (blue colored symbols) and background samples (green colored symbols). Strong positive correlations ($R^2 > 0.7$) confirm that these elements co-varied and likely originated from a common source.

Section S5. Interpretations of rare earth metal concentrations

Oddo-Harkin behavior

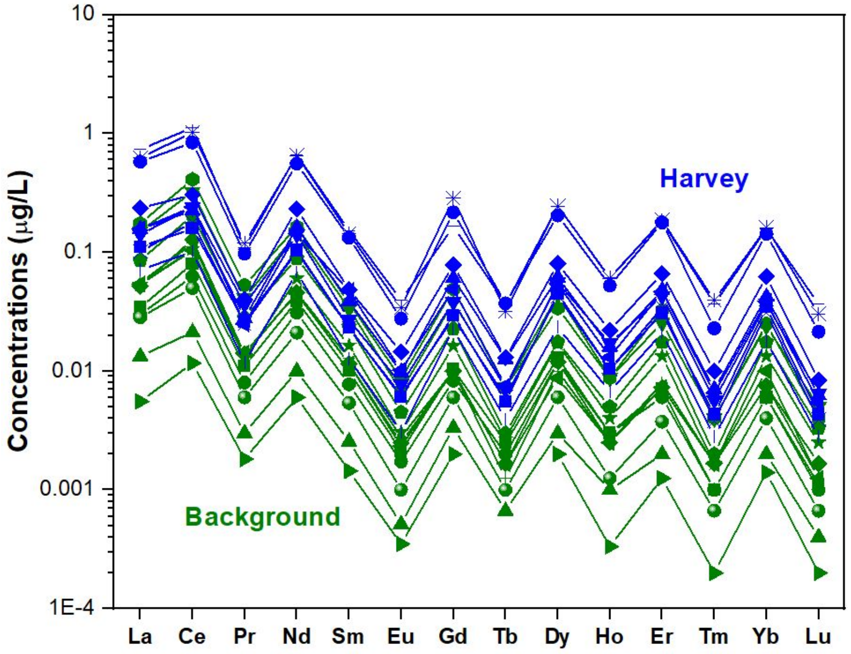

Figure S4. REE abundances for all Harvey remnants (blue color) and background samples (green color) followed Oddo-Harkins patterns (except for the samarium anomaly), similar to relevant crustal matrices such as local Houston soil, the North American Shale Composite,[1] and the Upper Continental Crust.[2]

Negligible cerium and europium anomalies

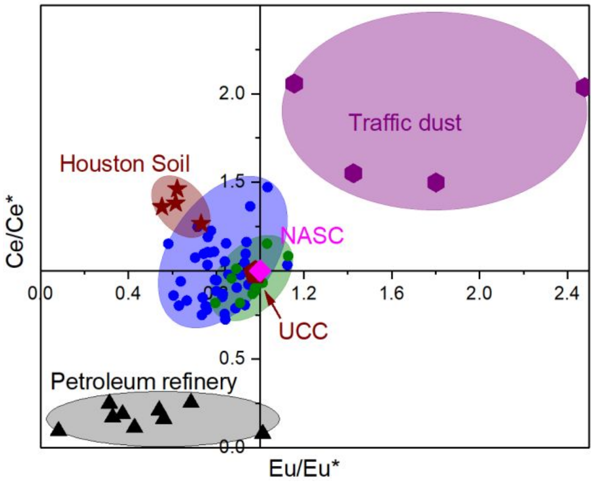

Figure S5. Ce and Eu anomalies showed REEs in Harvey samples as mixing from background and Houston soil regions and distanced itself from anthropogenic REEs (industrial/refinery sources[3] and traffic sources[4, 5]). The blue symbols denote Harvey floodwater remnants and the green symbols denote background data.

Section S6 Enrichment factors

Enrichment factors[6] of individual elements (X) in water samples were calculated referencing titanium (Ti) in Lake Houston background sample.[3,5]

$$Enrichment\ factor\ (X) = \frac{[X]_{sample} / [Ti]_{sample}}{[X]_{Lake\ Houston} / [Ti]_{Lake\ Houston}}$$

Titanium was chosen as the datum over other potential crustal reference elements such as aluminum and silicon because it is free of interferences from aluminosilicate (zeolite) sources that are widely employed in petroleum refining in Houston. Similarly, iron was avoided because ferrous metal working and recycling industries are also common in Houston. In other words, there are anthropogenic sources of Al, Si, Fe, and even Mn in Houston disallowing their use. Note that our research group has used Ti as the reference element in multiple previous publications related to metals in greater Houston[4, 5, 7-10] validating this choice. Lake Houston water was chosen as a regional background, because it integrates watershed-scale geochemical inputs under non-flood conditions and represents the most appropriate local aqueous baseline. Enrichment factors near unity suggest a similar concentration pattern in the water sample and background composition of Lake Houston. Enrichment factors near unity suggest a similar concentration pattern in the water sample and background composition of Lake Houston. Typically, only large variations in enrichment factors are amenable to strong interpretation, which is why the y-axis in Figure 4 of the main manuscript is depicted in logarithmic scale.

Another approach to express enrichment in geochemical investigations is through the geoaccumulation index (Igeo).[11, 12] However, this index is specifically designed for sediments and soils referenced to stable geochemical backgrounds and is not physically meaningful for dissolved metal(loids) in floodwaters subject to extreme dilution and irregular/transient mixing. Therefore, it was not pursued further in this manuscript.

Section S7. Yttrium to holmium ratios (Y/Ho)

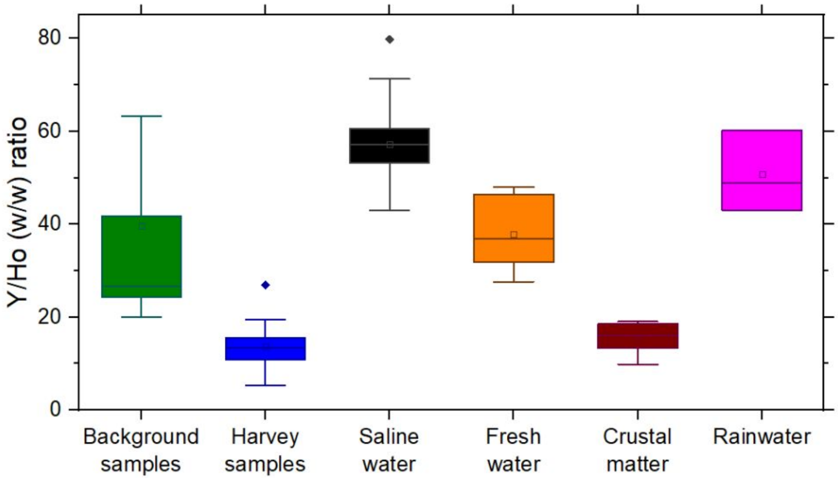

Figure S6. Y/Ho ratios for different matrices. Rainwater data taken from literature.[13-15]

Section S8. Score plots for principal components

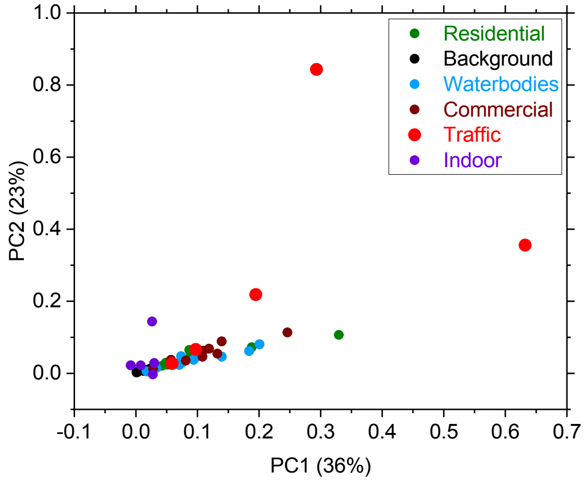

Figure S7. Score plot of samples for PC1 and PC2 showed that samples from near traffic sites were more influenced by PC2, inferring PC2 resembles a traffic source.

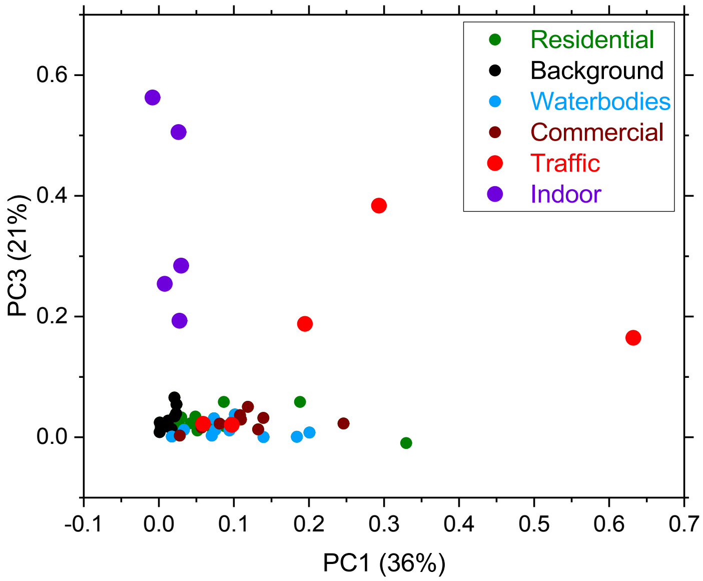

Figure S8. PC1 versus PC3 score plot showing separation and grouping of residential and commercial samples (violet color).

Section S9. Maps showing overall information on floodwater and background sampling locations.

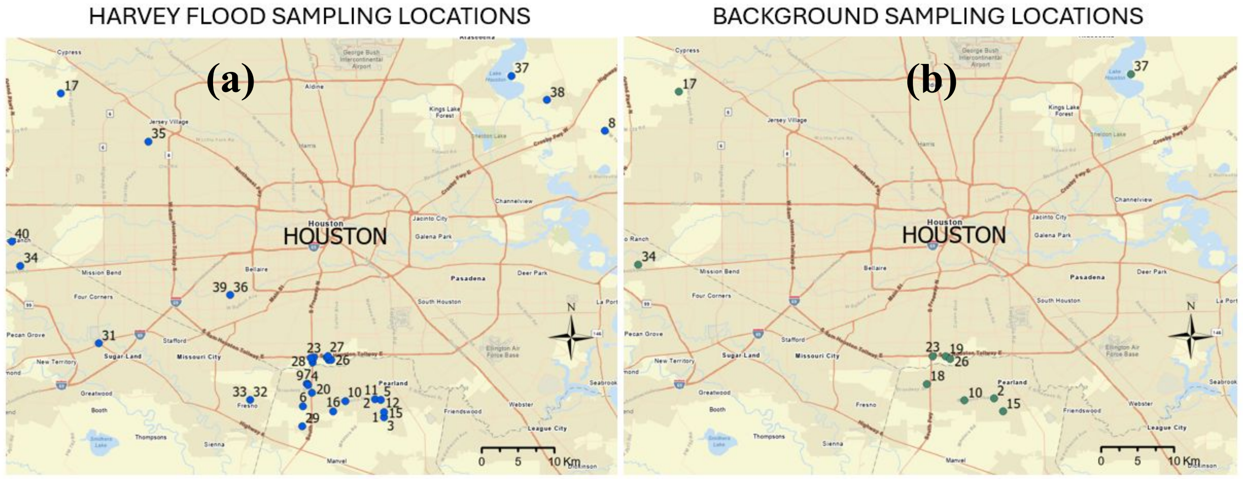

SI Figure S8. Overview of sampling locations in greater Houston. (a, left panel) Harvey floodwater remnant sampling locations. (b, right panel) Background sampling locations (under non-flooding conditions). This is only to provide a broad visual of our sampling locations within greater Houston. The main manuscript has a detailed cartographic description of the sampling locations.

REFERENCES

1. Gromet, L. P.; Dymek, R. F.; Haskin, L. A.; Korotev, R. L., The North-American Shale Composite - Its Compilation, Major and Trace-Element Characteristics. *Geochimica Et Cosmochimica Acta* **1984,** *48*, (12), 2469-2482.
2. Gaschnig, R. M.; Rudnick, R. L.; McDonough, W. F.; Kaufman, A. J.; Valley, J. W.; Hu, Z.; Gao, S.; Beck, M. L., Compositional evolution of the upper continental crust through time, as constrained by ancient glacial diamictites. *Geochimica Et Cosmochimica Acta* **2016,** *186*, 316-343.
3. Kulkarni, P.; Chellam, S.; Fraser, M. P., Lanthanum and lanthanides in atmospheric fine particles and their apportionment to refinery and petrochemical operations in Houston, TX. *Atmospheric Environment* **2006,** *40*, (3), 508-520.
4. Das, S.; Miller, B. V.; Prospero, J.; Chellam, S., Sr-Nd-Hf isotopic analysis of reference materials and natural and anthropogenic particulate matter sources: Implications for accurately tracing North African dust in complex urban atmospheres. *Talanta* **2022,** *241*, 123236.
5. Bozlaker, A.; Spada, N. J.; Fraser, M. P.; Chellam, S., Elemental characterization of $PM_{2.5}$ and PM_{10} emitted from light duty vehicles in the Washburn tunnel of Houston, Texas: Release of rhodium, palladium, and platinum. *Environmental Science & Technology* **2014,** *48*, (1), 54-62.
6. Varol, M.; Ustaoğlu, F.; Tokatlı, C., Metal pollution, eco-health risks and source apportionment in coastal sediments of Samsun, Türkiye: A receiving zone for the Kızılırmak and Yeşilırmak rivers. *Environmental Research* **2025,** *282*, 122113.
7. Das, S.; Miller, B. V.; Prospero, J. M.; Gaston, C. J.; Royer, H. M.; Blades, E.; Sealy, P.; Chellam, S., Coupling Sr–Nd–Hf Isotope Ratios and Elemental Analysis to Accurately Quantify North African Dust Contributions to $PM_{2.5}$ in a Complex Urban Atmosphere by Reducing Mineral Dust Collinearity. *Environmental Science & Technology* **2022,** *56*, (12), 7729-7740.
8. Williamson, K.; Das, S.; Ferro, A. R.; Chellam, S., Elemental composition of indoor and outdoor coarse particulate matter at an inner-city high school. *Atmospheric Environment* **2021,** *261*, 118559.
9. Das, S.; Chellam, S., Estimating light-duty vehicles' contributions to ambient $PM_{2.5}$ and PM_{10} at a near-highway urban elementary school via elemental characterization emphasizing Rhodium, Palladium, and Platinum. *Science of the Total Environment* **2020,** *747*, 141268.
10. Spada, N.; Bozlaker, A.; Chellam, S., Multi-elemental characterization of tunnel and road dusts in Houston, Texas using dynamic reaction cell-quadrupole-inductively coupled plasma–mass spectrometry: evidence for the release of Platinum group and anthropogenic metals from motor vehicles. *Analytica Chimica Acta* **2012,** *735*, 1-8.
11. Tokatlı, C.; Ustaoğlu, F.; Yazman, M. M.; Yüksel, B., Where rivers meet the sea: Source fingerprinting and health risk mapping of potentially hazardous elements in sediments from the Çanakkale Strait basin (Türkiye). *Marine Pollution Bulletin* **2026,** *222*, 118626.
12. Barbier, M., The Importance of Enrichment Factor (EF) and Geoaccumulation Index (Igeo) to Evaluate the Soil Contamination. *Journal of Geology & Geophysics* **2016,** *5*, (1), 1000237.
13. Iwashita, M.; Saito, A.; Arai, M.; Furusho, Y.; Shimamura, T., Determination of rare earth elements in rainwater collected in suburban Tokyo. *GEOCHEMICAL JOURNAL* **2011,** *45*, (3), 187-197.

14. Zhang, J.; Liu, C.-Q., Major and rare earth elements in rainwaters from Japan and East China Sea: Natural and anthropogenic sources. *Chemical Geology* **2004,** *209*, (3), 315-326.

15. Nozaki, Y.; Zhang, J.; Amakawa, H., The fractionation between Y and Ho in the marine environment. *Earth and Planetary Science Letters* **1997,** *148*, (1), 329-340.
